# Assessing land-use regulations for petrol stations in South Africa’s major cities

**DOI:** 10.4102/jamba.v17i1.1898

**Published:** 2025-08-29

**Authors:** Kwanele Qonono, Wilfred Lunga

**Affiliations:** 1 Department of Geography and Environmental Studies, Centre for Regional and Urban Innovation and Statistical Exploration (CRUISE), Faculty of Arts and Social Sciences, Stellenbosch University, Stellenbosch, South Africa; 2 Research Impact Division, Impact and Evaluation Unit, Human Sciences Research Council, Cape Town, South Africa; 3 Department of Developmental Capable Ethical State, Human Science Research Council, Pretoria, South Africa; 4 Disaster Management Training and Education Centre for Africa (DIMTEC), University of the Free State, Bloemfontein, South Africa; 5 African Centre for Disaster Studies (ACDS), North-West University, Potchefstroom, South Africa

**Keywords:** compliance, disaster risk reduction, environmental impact assessment, hazards, petrol stations, petroleum products

## Abstract

Noncompliance with locational guidelines for petrol station facilities in South Africa’s urban areas is widespread, posing significant disaster risks. Petrol stations store and handle flammable substances such as liquefied petroleum gas, hydrogen and biodiesel, making adherence to environmental impact assessment (EIA) safety parameters critical. This study evaluates the spatial distribution and compliance of petrol stations in Johannesburg, Cape Town and Durban with the 2002 EIA guidelines. Using a mixed-methods approach, geospatial mapping via Google Earth and ArcGIS alongside secondary qualitative analysis, the study assessed petrol station distances from sensitive land uses (residential, public institutions and critical infrastructure) and the spacing between petrol stations. The results reveal a significant degree of noncompliance with the EIA’s 100-m setback from sensitive uses and the 3-km minimum distance between petrol stations. In all three cities, petrol stations are often clustered within high-density urban cores, overlapping safety buffer zones and situated near residential and institutional structures. These spatial patterns indicate a regulatory gap in land-use enforcement and raise serious concerns about disaster preparedness and risk exposure in urban areas. The findings emphasise the urgent need to integrate disaster risk reduction (DRR) into urban land-use planning. While the study acknowledges that existing noncompliant petrol stations cannot be retrofitted easily, it recommends embedding DRR into future siting policies and calls for emergency preparedness measures at high-risk sites.

**Contribution:**

The study’s spatially grounded analysis of EIA compliance across multiple cities in South Africa offers an evidence-based framework to guide future policy on hazardous facility siting in disaster-prone urban contexts.

## Introduction

Despite the inherently hazardous nature of petrol stations because of their storage and handling of flammable and explosive substances, there is limited empirical research on their disaster risk potential and regulatory compliance in South Africa’s urban landscape (Qonono 2024). International evidence highlights the disastrous impacts of such hazards, yet local studies have largely focused on fuel pricing, economic benefits and health effects (Bennett 1990; Hadland 2002; Moolla & Curtis 2013; Moolla, Curtis & Knight 2015; Qonono 2019). Globally, mishandled petroleum products have caused over 2.3 million deaths and $4.5 billion in property losses (WHO 2004). The 2022 liquefied petroleum gas (LPG) tanker explosion in Boksburg, South Africa, which killed over 41 people, exemplifies the extreme risks associated with transporting and storing petroleum products (Özbakır 2024).

Hazards are defined as events that may cause injury, death or environmental damage (UNDRR 2023) – are amplified at petrol stations. These sites present various risks, including fires, chemical exposure, pollution, workplace injuries and criminal threats. Petrol is highly flammable and can ignite easily, making strict adherence to safety protocols essential (IEA 2022; Kumar & Bansal 2023). Vapours like benzene, found in petrol and diesel, are linked to cancer, organ damage and reduced fertility, posing public health concerns (Maung et al. 2022; World Bank 2017). Spills from these facilities can contaminate soil and groundwater – one gallon of gasoline can pollute up to one million gallons of water (Mohammed, Ibrahim & Patel 2014; NACS 2022).

Operational hazards also include slips, falls and physical injuries caused by fuel spills and poor handling practices (Mäkká et al. 2023; Spencer 2004). Additionally, petrol stations are often targeted for theft and violence because of the presence of cash and valuable goods, necessitating robust security measures (Hanekom 2001).

Modern petrol stations range from simple service points to larger complexes offering diesel, LPG, natural gas, hydrogen, biodiesel and convenience stores (ARA 2009; Ayodele 2011; Genovese 2004). Although their primary function remains fuel retailing, evolving demand and environmental regulations have increased the complexity and risk of their operation (Kumar & Bansal 2023; World Bank 2017).

Numerous global incidents demonstrate the severe impacts of petrol station fires. In France, over 270 fires were reported between 1958 and 2007 (Ministry of Ecology 2009). The United States recorded an annual average of 5020 fires at service stations between 2004 and 2008, resulting in fatalities and millions in damages (Qonono 2019; United States National Fire Protection Association [NFPA] 2011). In China, an explosion in Shaanxi Province caused injuries and extensive environmental damage (Newsflare 2018; Qonono 2019).

Africa has witnessed equally alarming events. Between 2007 and 2014, Ghana experienced 11 LPG-related accidents resulting in 39 deaths (Bokpe 2015). A 2015 Accra explosion killed over 150 people (Al Jazeera 2017). Similar disasters have occurred in Nigeria, Uganda and South Africa, including the Cato Ridge and Mvoti Ultra City explosions (Al Jazeera 2023; Anadolu Agency 2024; ENCA 2015; Savides 2016).

Urban proximity increases disaster risk. In Tehran, 480 petrol station fires occurred between 2002 and 2006 (Nouri, Omidvari & Tehrani 2009), and Cape Town recorded 268 incidents between 2009 and 2017 (City of Cape Town 2018; Qonono 2019). These incidents often result from weak land-use planning and inadequate enforcement of zoning laws (Özbakır 2024; Qonono 2019; United States National Fire Protection Association [NFPA] 2011). When petrol stations are sited near residential areas, schools or commercial hubs, community vulnerability escalates (UNISDR 2009; Zhou & Liu 2011).

Sutanta (2012) argues that land-use planning plays a critical role in disaster risk reduction (DRR) by (1) preventing development in hazardous zones, (2) categorising land by risk exposure and (3) criminalising noncompliant development. Pelling (2003) similarly notes that poor planning often creates risk rather than mitigating it.

This study contributes to the DRR and land-use planning discourse in South Africa by integrating spatial analysis with regulatory evaluation. It highlights systemic noncompliance with EIA guidelines for hazardous facilities and calls for stronger policy enforcement. The findings support proactive risk management and advocate for improved preparedness strategies, offering a spatially grounded framework for safer urban development across other rapidly growing cities.

### Research aims and objectives

This paper evaluates petrol stations compliance with land-use guidelines in South Africa’s three metropolitan areas: Johannesburg, Cape Town and Durban. The paper assesses the distances between petrol stations, residential houses, public institutions and other critical facilities in the urban core of South Africa’s metropolitans. The specific objectives were to:

1. Identify and map petrol station facilities in Johannesburg, Cape Town and Durban’s urban area.
2. Determine the land-use type around these petrol stations and the extent to which petrol station positioning complies with EIA guidelines of 2002.
3. Recommend appropriate risk reduction strategies and preparedness to face emergency conditions of gas station fire facilities.

## Methods and design

### Study area

The study focuses on three major urban centres in South Africa: Johannesburg, Cape Town and Durban. These cities represent the country’s largest metropolitan regions, characterised by dense commercial and residential development, high vehicle usage and growing demand for fuel. Johannesburg, located in Gauteng Province, is South Africa’s economic hub with the highest GDP per capita. Cape Town, the legislative capital, is situated in the Western Cape and is known for its mixed land-use patterns and tourism-driven economy. Durban, a coastal city in KwaZulu-Natal, hosts two major oil refineries and serves as a key logistics gateway through its port. These urban areas were selected for their size, economic activity and strategic importance in the national fuel supply network.

### Data collection approach

A mixed-methods approach was adopted, integrating geospatial data analysis and secondary data analysis to assess the disaster risks posed by petrol stations. Mixed methods research allows for a comprehensive examination of complex research issues by combining qualitative and quantitative data, thus mitigating the limitations of single-method approaches (Creswell & Clark 2018). The integration of these methods enhances analytical depth, ensuring robust risk assessment and spatial representation of petrol station facilities (Tashakkori & Teddlie 2010).

### Secondary data

Secondary data analysis was employed to evaluate disaster risks associated with petrol stations, utilising datasets from government reports, environmental impact assessments (EIAs) and accident records from regulatory agencies. These sources provided critical insights into historical incidents, environmental hazards and compliance with safety regulations (Paton 2020). The geospatial data analysis focused on mapping and analysing the spatial distribution of petrol stations in Johannesburg, Cape Town and Durban. This involved identifying the geographic coordinates (GPS points) of petrol station facilities to assess their proximity to high-risk areas such as residential zones, industrial hubs and transportation networks.

### Geospatial data

**FIGURE 1:**
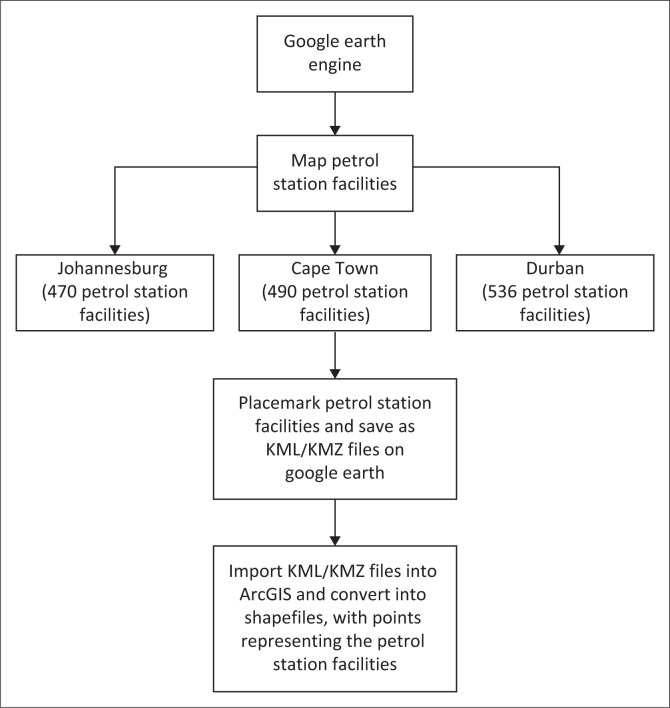
Method to identify and map petrol station facilities in the cities.

Petrol station facilities were identified using Google Earth, where each facility was marked with a placemark. The locations were saved as KML or KMZ files and subsequently imported into ArcGIS for geospatial analysis. These files were converted into shapefiles, representing petrol station locations as point data layers. This methodology facilitated precise spatial visualisation and risk assessment. The analysis revealed a total of 470 petrol station facilities mapped in Johannesburg, 490 in Cape Town and 536 in Durban (Figure 1). These mapped facilities were analysed in relation to urban infrastructure, land-use patterns and potential hazard zones, contributing to a spatially explicit risk assessment framework (Goodchild 2022). The integration of geospatial techniques with secondary data analysis provided a comprehensive understanding of potential disaster risks posed by petrol stations in major South African cities.

### Data analysis

The study employed a desktop-based geospatial analysis approach using GIS tools (Google Earth and ArcGIS) to identify, map and analyse the spatial distribution of petrol station facilities in Johannesburg, Cape Town and Durban. The analysis focused on evaluating compliance with two key EIA 2002 guidelines: (1) maintaining a 100-m setback from sensitive land uses such as residential areas, schools and hospitals and (2) ensuring a minimum 3-km spacing between petrol stations in urban and built-up areas. Petrol stations were geo-referenced and overlaid with spatial data to assess their proximity to restricted areas and to one another. This method allowed the researcher to visualise clustering patterns and detect land-use conflicts. While comprehensive in spatial terms, the analysis was limited to secondary data and did not include input from local officials on approval processes or historical compliance trends.

### Land-use planning and risk generation: Location of petrol station facilities

The location of petrol station facilities in urban areas across many African countries is typically governed by formal planning frameworks such as structure plans or land-use development plans (Taylor, Sichinsambwe & Chansa 2016). These guidelines set specific locational requirements intended to mitigate risks associated with hazardous materials. Local authorities are tasked with enforcing these plans, often advised by urban planners and environmental consultants (Taylor et al. 2016). Despite this, many cities exhibit a proliferation of petrol stations that fail to comply with these guidelines, increasing exposure to environmental, health and fire risks.

In Nigeria, Emakoji and Otah (2019) observed widespread violations in Afikpo, where nearly half of the gas stations were located too close to healthcare centres and roadways, while 66.7% did not maintain the required 400-m spacing between stations. Similarly, in Ilorin Metropolis, Ibrahim et al. (2019) noted poor adherence to safety protocols and low awareness of health risks among vendors using aboveground storage tanks. In Maiduguri and Jere, Abdullahi and Dawha (2015) found a dangerous concentration of petrol stations near residential areas, with many established without consideration for siting guidelines, posing environmental and health threats.

Adedeji et al. (2022) highlighted the indiscriminate placement of petrol stations in Abeokuta, often within residential, commercial and educational zones. These stations frequently lacked firefighting systems and failed to meet the 450-m safety buffer. Similarly, Abiola and Oyinloye (2021) documented cumulative noncompliance in Ife Central, where most petrol stations were located less than 30 m from homes and roads. Ulakpa, Ulakpa and Eyankware (2022) echoed these concerns, reporting widespread violations of basic planning setbacks across Nigeria.

In Ibadan, Kehinde, Bolanle and Ayomide (2021) reported that only 32.86% of stations adhered to international spacing standards, and a mere 7.14% maintained a 50-m buffer from critical public buildings. The remaining 92.86% were noncompliant, increasing the risk to nearby residents and institutions. Similarly, Taylor et al. (2016) noted excessive clustering of petrol stations in Kitwe’s Central Business District, Zambia, with little regard for environmental safety.

Ghana faces similar challenges. Damnyag and Aazagreyir (2020) found that most of the 37 surveyed stations had a cluster distribution along major roads, often violating spacing standards. They called for stricter regulatory enforcement. In Paga, Douti et al. (2019) found petrol stations sited near residences and places of public assembly, posing significant health and safety hazards. David et al. (2018) emphasised that retail fuel station locations have become increasingly haphazard, despite their high sensitivity to location-based risks.

Campion and Essel (2013) examined the EIA process in Ghana and found inconsistent application across projects involving filling stations. A lack of coordination among environmental authorities further complicated enforcement and oversight.

In Uganda, Wadembere and Apaco (2020) assessed spatial risk levels near petrol stations and concluded that buildings within 50 m were at the highest risk of fire-related disasters, while those within 100 m still faced moderate risk. Similarly, Qonono (2024), studying Stellenbosch in South Africa, found that many petrol stations were sited in violation of EIA (2002) guidelines, particularly regarding minimum distance from residential areas and spacing between stations.

Across these cases, a common theme emerges: despite the existence of national planning standards, enforcement is weak, and noncompliance is widespread. This failure to align land-use planning with disaster risk considerations creates environments of heightened vulnerability, particularly in densely populated urban centres. Table 1 shows guidelines for the location of petrol stations in Nigeria, Ghana, Uganda, Zambia and South Africa.

**TABLE 1:** Guidelines for the location of petrol stations in Nigeria, Ghana, Uganda, Zambia and South Africa.

Countries	Guidelines	Source
Nigeria	400 m minimum distance to other stations located on the same roadside. 15 m distance from the road. 100 m from the health care facilities. 50 m distance between one petrol station and the nearest residential building. 90 m distance between one petrol station and the nearest place of public assembly.	Department of Petroleum Resource (DPR), 2007, *Procedure guide for grant of and approval to construct and operate petroleum products retail outlets*, Issued by DPR, Ministry of Petroleum Resources in Nigeria.
Ghana	15 m distance from buildings. 10 m distance from other filling stations.	Abu Abdulai, I., Awal Abubakari, M. & Juah M-Buu File, D., 2024, *Siting of fuel stations within residential areas in Ghanaian cities: Perceptions of residents in Wa on fire disaster risks*, viewed 22 September 2024, from https://doi.org/10.1016/j.heliyon.2024.e29964
Uganda	0–100 m distance of buildings from fuel stations is considered very high risk. 50 m – 250 m distance of buildings from fuel stations is considered very high risk. 150 m – 500 m distance between the building and the fuel stations is considered a moderate risk level. Buildings 300 m – 500 m from fuel stations are considered low risk. Buildings more than 500 m from the fuel stations are considered very low-risk	Wadembere, I. & Apaco, J., 2020, ‘Urban spatial risk assessment of fire from fuelling stations on buildings case study: Lubaga Division, Kampala City, Uganda’, *Journal of Building Construction and Planning Research* 8, 57–72. https://doi.org/10.4236/jbcprt.2020.810
Zambia	50 m away from residential and private buildings. 40 m buffer zone of open space or front area for the road. 500 m away from the nearest petrol filling station. 100 m away from water bodies.	Energy Regulation Board (ERB), 2015, *Guidelines for sitting filling stations*, 1st Revision.
South Africa	100 m away from residential properties, schools or hospitals unless it can be clearly demonstrated that there will be no significant noise, visual intrusion, safety concerns or fumes and smells. Within 3 km of an existing filling station in an urban, built-up or residential. Within 25 km driving distance of an existing filling station in other instances, such as in rural areas and along highways and national roads) or Within a sensitive area such as wetlands, alongside rivers, etc.	Environmental Impact Assessment (EIA), 2002, *Guideline for the construction and upgrade of filling stations and associated tank installations: Agriculture, conservation, environment and land affairs, Gauteng province*, viewed 22 October 2024, from http://home.intekom.com/salbu/a_R21/FillingStationsGuidelines.html.

Noncompliance with locational requirements of the petrol stations is not uniquely African. There are several other cases of such behaviour elsewhere (Nieminen 2005). Globally, many countries do not seem to adhere to the requirements for the location of petrol station facilities. Numerous organisations appear to be issuing multiple rules and regulations covering the management of petrol station facilities, thus creating confusion among those involved in the process, including regulatory authorities and the importance of their rules (Nieminen 2005; Qonono 2019). Failing to comply with petrol stations’ locational requirements jeopardises people’s safety and proximal development.

Zoning and land-use regulations for filling stations in South Africa vary across metropolitan areas, reflecting localised governance frameworks and spatial priorities. In Cape Town, the Municipal Planning By-law (2015) designates fuel stations within ‘Local Business 2’ and ‘Risk Industry’ zones as ‘Service station & motor repair garage’ (City of Cape Town 2015). The updated Zoning Scheme By-law (2022) introduces a dedicated ‘Service Station (SS)’ zone, accommodating fuel retail, garages and ancillary services. Development parameters include a floor factor of 1.0, 75% maximum coverage, an 8 m height limit and a 3 m street building line. Access is restricted to two vehicular crossings, or three where adjacent road widths exceed 30 m and 2.1 m screening walls are required for storage and repair areas (City of Cape Town 2022).

In eThekwini (Durban), the Land Use Scheme under the Municipal Planning By-law (2016, amended 2021) allows fuel stations within ‘Fuelling and Service Station’ zones, subject to comparable design constraints, including setbacks and screening. However, additional regulatory requirements apply, including rezoning or consent use processes, traffic impact assessments (TIA) and compliance with NEMA and hazardous substance regulations (eThekwini Municipality 2021). In contrast, Gauteng Province applies provincial siting guidelines aligned with the Land Use Planning Act (LUPA), emphasising spatial separation. Minimum distances of 3 km (urban) and 25 km (rural) between stations are required to limit market saturation and enhance safety. Environmental Impact Assessments are compulsory where stations are within 100 m of sensitive receptors such as schools, residences or healthcare facilities (Gauteng Department of Roads and Transport 2009). Collectively, Cape Town and Durban exhibit comprehensive, zoning-driven regulatory models with technical consistency, while Gauteng adopts a precautionary, distance-based approach prioritising spatial planning and environmental protection.

### Ethical considerations

Ethical clearance to conduct this study was obtained from the Stellenbosch University Research Ethics Committee: Social, Behavioural and Education Research (REC: SBE) (No. 32003). Following review, the committee granted the study an ethics exemption.

## Results

### Findings relating to research objectives

The results are presented according to each research objective. Firstly, the spatial distribution of petrol station facilities is identified and mapped across Johannesburg, Cape Town and Durban. Secondly, the land-use types surrounding these petrol stations are determined, specifically examining compliance with the Environmental Impact Assessment guidelines regarding proximity to sensitive areas such as residential zones, schools and hospitals. Thirdly, recommendations for appropriate DRR strategies are provided, with particular emphasis on preparedness measures to address fire and explosion risks at petrol station facilities in urban areas.

### Distribution of petrol station facilities in South Africa

**FIGURE 2:**
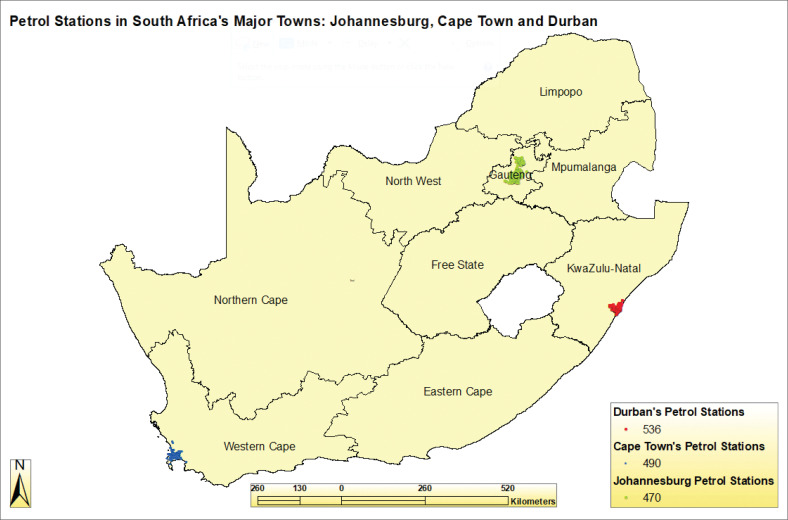
Petrol station facilities in Johannesburg, Cape Town and Durban.

South Africa has approximately 4600 petrol station facilities and Africa’s second-largest oil refining capacity (Smith 2022). In 2018, South Africa produced 520 000 barrels a day (1 barrel equals 159 l), resulting in (30 190 200 000 [30 bn] litres per year). Johannesburg, Cape Town and Durban combined consumed more than 20bn litres, about 67% of the total production per year (Smith 2022). A total of 470 petrol stations were mapped in Johannesburg, 490 in Cape Town and 536 in Durban (Figure 2).

### Land-use type around these petrol stations

#### Johannesburg

**FIGURE 3:**
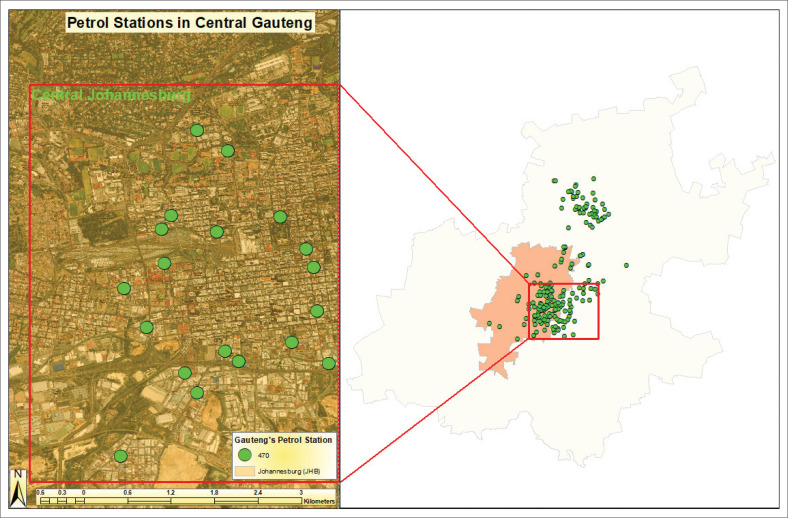
Petrol station facilities in central Johannesburg.

Johannesburg, South Africa’s economic hub, holds the highest GDP per capita and growth rate among the country’s cities. With an average combined fuel volume of 320 kL per month per site in 2021, it is the most attractive market for fuel investment (Smith 2022). Since 2020, 20 new petrol stations have been added, and 56 are currently under construction, bringing the total to approximately 470 facilities. This growth reflects Johannesburg’s increasing fuel demand driven by dense commercial and residential activities. Investment success in this competitive market depends on identifying optimal locations. Figure 3 maps petrol station facilities in central Gauteng, highlighting the dense clustering of stations – particularly in central Johannesburg – marked by green dots. The red box outlines this high-demand core, while the surrounding green dots across the broader Gauteng region indicate a widespread presence of petrol stations beyond the central urban area, aligning with transportation routes and expanding urban sprawl.

Within the zoomed-in section of central Johannesburg, petrol station facilities are spread relatively evenly throughout the area, with no significant gaps in coverage. The petrol station facilities are located near major roads and highways, which is typical for urban planning strategies, ensuring that vehicles have easy access to fuel. The high density of petrol station facilities in this urban environment suggests careful planning to balance accessibility with safety. However, the concentration of petrol station facilities in such a busy area may raise concerns about safety regulations, especially those related to the proximity to residential or commercial buildings and the risk of accidents, pollution or other hazards. The proximity of petrol station facilities ensures easy access but could pose safety challenges because of the potential risks associated with fuel handling in dense urban settings. This concentration also points to the importance of regulatory compliance, ensuring that all stations adhere to safety standards, particularly regarding their distance from other infrastructure or sensitive areas.

#### Cape Town

**FIGURE 4:**
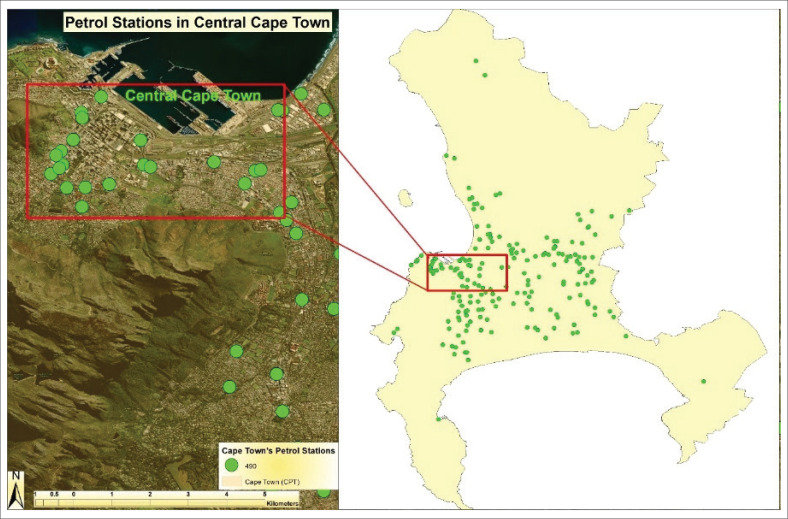
Petrol station facilities in central Cape Town.

Cape Town ranks second after Johannesburg in fuel demand, with an average fuel volume of 297 kL per month per site (Smith 2022). The city has around 490 operational petrol station facilities. However, the pace of new construction has slowed – from 15 new stations in 2019 to only eight in 2021 – because of limited available land and lengthy municipal approval processes. Fierce competition for land has shifted investor interest toward acquiring or upgrading existing stations, especially among independent and unbranded operators. Figure 4 maps petrol station facilities in central Cape Town, showing green dots clustered around the Central Business District (CBD) and major roads. These facilities serve a high-density urban population and reflect strong commercial activity. The red box highlights central Cape Town, while the wider map shows a more dispersed pattern in outlying areas. This distribution indicates that although fuel access is widespread, development constraints are increasingly shaping the spatial distribution of new stations.

#### Durban

**FIGURE 5:**
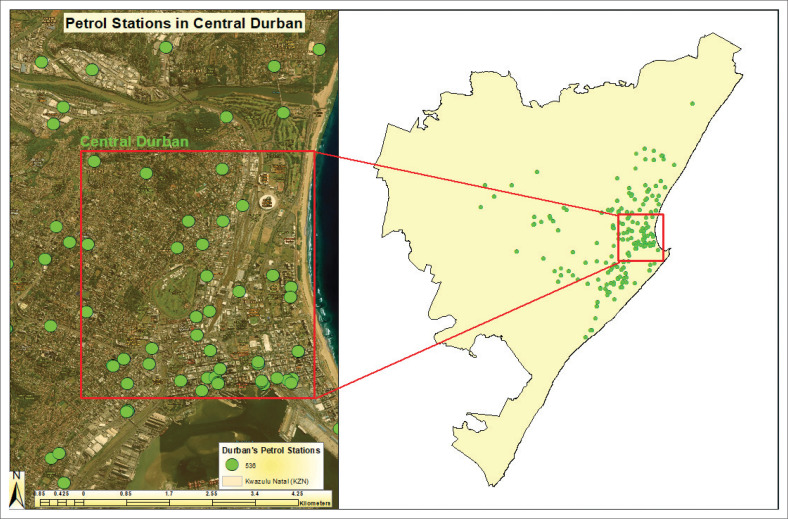
Petrol station facilities in central Durban.

Durban hosts approximately 536 petrol station facilities and benefits from being home to two of South Africa’s largest oil refineries. It also serves as a key port for importing crude and refined fuels, and it connects to the inland market via major fuel pipelines (Smith 2022). Unlike Cape Town, Durban still offers opportunities for new developments and acquisitions, attracting investors seeking market entry. The city’s high diesel volumes contribute to strong average fuel throughput per site, similar to Cape Town. Figure 5 illustrates the dense concentration of petrol stations in central Durban, particularly along key roads and urban corridors. Green dots mark the petrol station locations, with the red box highlighting central Durban’s urban core. Facilities are also visible in the broader Durban metropolitan area, especially along coastal routes and high-traffic zones. This spread reflects both the city’s logistical significance and growing fuel demand from residents, industries and passing motorists.

### Compliance with the Environmental Impact Assessment guidelines

The 2002 EIA guidelines were developed to ensure the safe siting of petrol station facilities and minimise risks to surrounding developments. Though developed in Gauteng, these guidelines were affirmed by the Constitutional Court and apply nationally (EIA 2002). They require stations to be at least 100 m from residential buildings, schools or hospitals – unless no safety or environmental concerns can be demonstrated – and prohibit new stations within 3 km of existing ones in urban areas or 25 km in rural settings. These standards are grounded in global best practices and South Africa’s environmental legislation under Environmental Conservation Act (ECA) and National Environmental Management Act (NEMA).

#### Compliance of petrol station facilities in central Johannesburg with a 100 m radius

**FIGURE 6:**
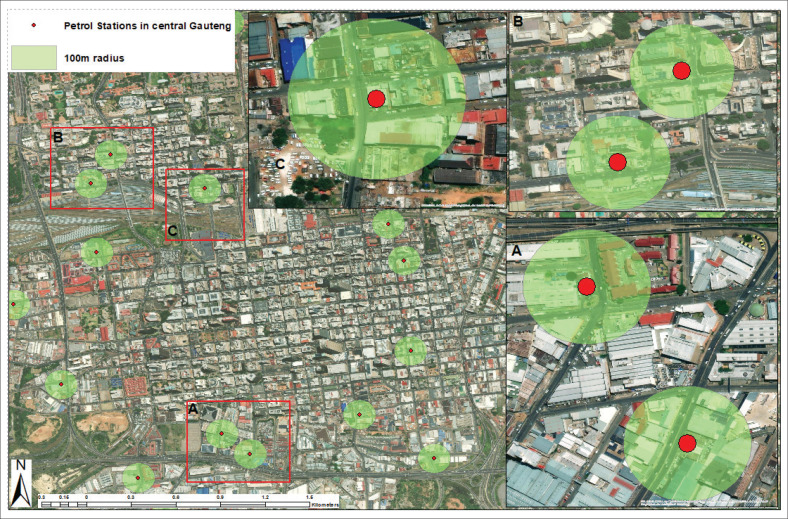
Petrol station facilities within a 100 m radius of central Johannesburg.

Figure 6 shows the locations of petrol station facilities in central Gauteng, marked by red dots, and a 100-m radius (highlighted in green) around each petrol station facility. The map shows a significant clustering of petrol station facilities in some areas. For example, in areas marked as A and B, multiple petrol station facilities are located close to each other within a 100-m radius (Figure 6). This concentration may indicate a high central positioning within busy urban areas. In these clusters, the proximity of petrol station facilities raises potential issues related to urban planning, traffic congestion and safety. The map also highlights how most petrol station facilities are concentrated in the central metropolitan area of Gauteng, which appears to be a densely populated and developed region. The proximity of buildings and infrastructure within the green 100 m radius suggests that these stations are located near various structures, such as commercial and residential buildings. The central location of these petrol station facilities poses potential risks to nearby inhabitants and businesses if the necessary safety distances, as per regulations, are not met.

Several petrol station facilities appear near major roads or intersections, providing easy access to motorists. These strategic placements are typical for ensuring high visibility and customer convenience in busy areas, but this could also contribute to traffic build-up in certain areas. In areas where multiple petrol stations are clustered (such as B and C), the 100-m safety radius of one station may overlap with others, increasing the potential risk of accidents or environmental hazards, especially if proper safety measures are not in place. Some petrol station facilities are located away from the cluster, spread across the urban region, but within the 100-m safety radius. These petrol station facilities might be better situated in terms of adhering to safety regulations, as they are more isolated and less likely to face the risks associated with clustered stations. The proximity of petrol station facilities within densely populated areas may raise concerns about fire hazards, environmental pollution and the potential impact on residential areas or businesses. The 100 m radius zones marked on the map in Figure 6 can serve as a reference to check whether these stations comply with local safety regulations regarding their distance from other buildings and infrastructure. This map demonstrates the complex nature of petrol station facility placement within an urban context and highlights potential risks related to clustering and proximity to critical infrastructure.

#### Compliance of petrol station facilities in central Cape Town with a 100 m radius

**FIGURE 7:**
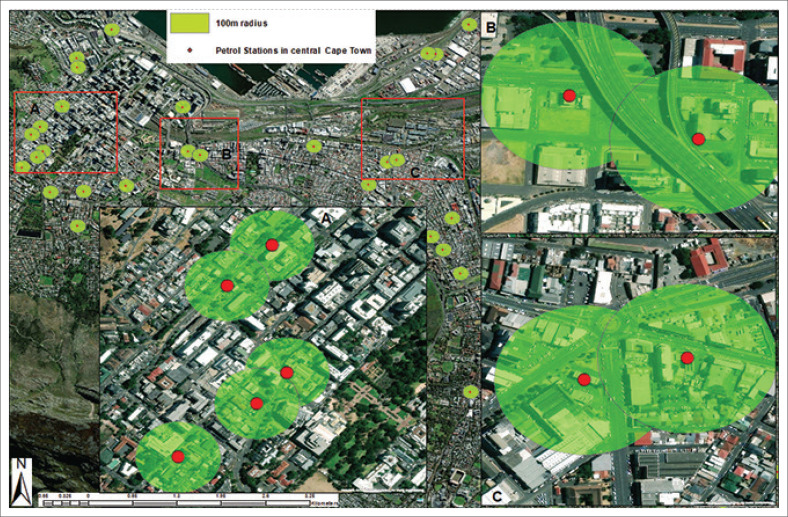
Petrol station facilities within a 100 m radius of central Cape Town.

Figure 7 illustrates the distribution of petrol station facilities within central Cape Town, emphasising a 100-m radius around each station. The large map at the top highlights several petrol station facilities within Cape Town’s central district. The green circles represent a 100-m radius around each petrol station facility, helping visualise proximity and potential overlaps in service areas. The petrol station facilities are marked by red dots, primarily located in areas of high urban density. The 100-m radius suggests some overlap in service areas, particularly in highly urbanised sections. The 100-m safety radius of one station overlaps with others, with residential and commercial areas increasing the risk of environmental hazards, especially if proper safety measures are not in place. These petrol station facilities do not comply with a 3 km radius stipulated in the EIA (2002). Area A highlights an intense concentration of stations in the city centre.

#### Compliance of petrol station facilities in central Durban with a 100 m radius

**FIGURE 8:**
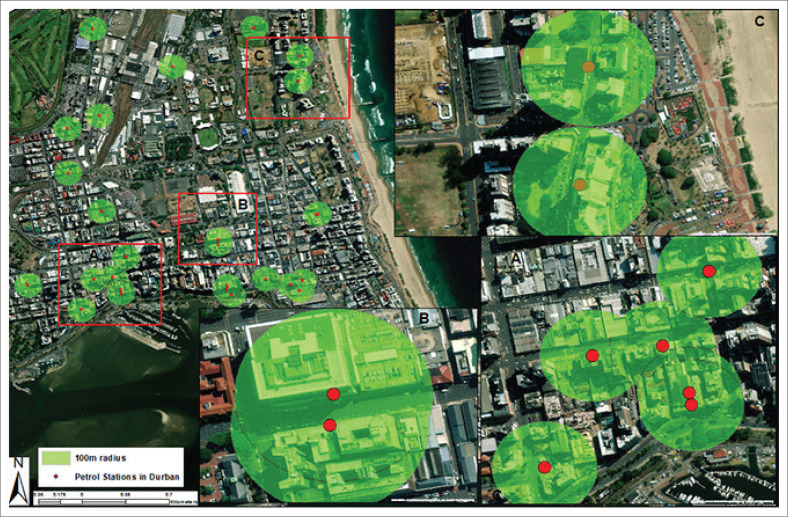
Petrol station facilities within a 100 m radius of Durban.

Figure 8 shows the locations of petrol station facilities in Durban, with green circles indicating a 100-m radius around each station. Multiple petrol station facilities within proximity overlap, suggesting they are closer than 100 m apart, violating the 100-m radius rule. Similar to Johannesburg and Cape Town, in areas with dense clustering of petrol station facilities (such as A and B), there appears to be noncompliance with the 100-m radius rule because of significant overlap. In less dense areas, such as C and other outlying stations, the 100-m radius seems more spread out, indicating non-overlap. However, residential and commercial areas are still within the 100-m radius and 3-km radius between petrol station facilities, which is non-compliant in the three cities.

## Discussion

Recent studies on compliance of petrol station facilities with land-use guidelines and siting requirements suggest more significant levels of non-compliance, particularly in Nigeria (Abdullahi & Dawha 2015; Abiola & Oyinloye 2021; Adedeji et al. 2022; Ajman et al. 2021; Emakoji & Otah 2019; Ibrahim et al. 2019; Kehinde et al. 2021; Ulakpa et al. 2022), Ghana (Bosco & Aazagreyir 2020; David et al. 2018; Douti et al. 2019), Uganda (Wadembere & Apaco 2020), Zambia (Taylor et al. 2016) and South Africa (Qonono 2024). This study’s results also show greater noncompliance levels of petrol station facilities in South Africa’s most populated urban areas: Johannesburg, Cape Town and Durban.

Most African countries have strict regulations for the location of petrol station facilities, with variations, commonalities and specific distances from residential or other important buildings. For example, Nigeria and Ghana have a minimum distance of 15 m from buildings (Dongzagla et al. 2023; DPR 2007), while Uganda, Zambia and South Africa have varying distance requirements based on the assessed risk or location. Each country has guidelines prioritising public safety and environmental protection (EIA 2002; ERB 2015; Wadembere & Apaco 2020). Zambia, South Africa and Uganda identify areas with very high risk or sensitive zones, such as residential areas, hospitals and water bodies, requiring specific considerations. These countries have guidelines prioritising public safety and environmental protection. Uganda and South Africa incorporate a tiered risk-based approach, separating high, moderate and low risks depending on the distance from fuel stations. In its guidelines, South Africa accounts for the type of area (urban, sensitive or near highways) to determine the required distance from petrol station facilities (EIA 2002).

Nigeria mandates distances for multiple scenarios (e.g. 400 m between stations on the same road, 50 m from residential buildings, 10 m from other filling stations, etc.) (DPR 2007). In contrast, it specifies 15 m from buildings and 10 m from different petrol station facilities without further subdivision risk levels. On the other hand, Uganda categorises distances into high (0–100 m), moderate (100 m – 500 m) and low (more than 500 m) risk levels (Wadembere & Apaco 2020). Zambia requires a minimum of 500 m away from petrol station facilities and other areas, which is one of the more restrictive requirements (ERB 2015). South Africa’s EIA guidelines include 100 m away from residential properties, schools or hospitals, offering more contextually specific rules than other countries (EIA 2002). South Africa goes beyond specifying distances, addressing potential concerns such as noise, safety from fumes and environmental and health risks. It also has more detailed provisions for urban and rural contexts, including distance from sensitive areas (EIA 2002). Notably, Zambia has a 500 m requirement away from the nearest petrol station, significantly larger than other countries’ requirements (ERB 2015). Nigeria has specific regulations for distances between stations on the same road, which aim to avoid clustering (DPR 2007).

These guidelines aim to mitigate risks associated with petrol stations, though the degree of strictness and focus differ across the regions. However, the level of noncompliance with these guidelines, particularly the EIA guidelines in the case of South Africa, is shocking (EIA 2002). In all three of South Africa’s significant towns, multiple petrol station facilities are located close to each other within a 100-m radius, and the 3-km radius parameter between petrol station facilities is completely disregarded. The proximity of petrol station facilities to where people live, properties and to each other raises potential issues related to urban planning and DRR as approached in siloes, with the risk of petrol station facilities less considered and the safety of the surrounding development. This also suggests a lack of oversight from local authorities in South Africa. The location of petrol station facilities poses potential risks to nearby inhabitants and businesses if the necessary safety distances, as per regulations, are not adhered to. The 2022 Boksburg LPG tanker blast on December 24 underscores the dangerous aspect of LPG (a petroleum product), including its handling installations, among other cases. Hence, compliance is essential when locating a petrol station facility.

### Recommendation

Noncompliance with location guidelines of the petrol station facilities in South Africa’s urban areas is on a grand scale. Pelling (2003:ii) argued that ‘disasters can result in failed development, but failures in development planning can also lead to disaster risk’. A typical example is the development planning failures regarding compliance with siting petrol station facilities in South Africa and elsewhere. Disaster risk reduction should be embedded in land-use development planning to protect people and their environment from hazardous materials and proactively manage risks rather than disasters (Britton & Lindsey 1995).

An integrated regulatory framework is essential to harmonise municipal zoning schemes with environmental and safety legislation, thereby addressing fragmented approval processes. Such a unified approach would ensure that land-use compatibility, environmental protection and public safety are jointly considered during site assessments (City of Cape Town 2015, 2022; eThekwini Municipality 2021; Gauteng Department of Roads and Transport 2009). A critical gap in current practice is the insufficient attention to fire and hazard risk assessments during pre-approval processes, which often prioritise traffic and environmental impacts while overlooking immediate safety risks. Comprehensive hazard evaluations should therefore be mandated prior to site approval.

To support spatial planning and prevent over-concentration, a real-time, GIS-enabled registry of all fuel stations and their zoning statuses should be developed. This tool would enhance regulatory oversight and assist municipalities in enforcing minimum separation guidelines. Mandatory public consultation should also be institutionalised for all new fuel station applications. Community input, particularly in densely populated areas, can provide valuable insights into local risks, such as proximity to residences, schools and healthcare facilities, thereby enhancing procedural transparency and social accountability. Finally, regulatory enforcement must be strengthened. A system of graduated penalties, including substantial fines and operational suspensions, should be implemented for non-compliance with zoning or environmental conditions. Post-approval monitoring is equally critical, ensuring that fuel stations continue to meet safety and planning standards as urban contexts evolve.

It may be too late for the already installed petrol station facilities to comply with EIA 2002 locational guidelines. Planned new petrol station facilities should adhere to the EIA guidelines. All that is required for the noncompliant petrol station facilities with EIA guidelines is to prepare for a possible disaster or emergency.

Disaster preparedness can help to predict and effectively respond to the effects of likely, imminent or present hazard events or situations (Qonono 2019; UNISDR 2008), while land-use planning can help to mitigate catastrophes and minimise risk (Qonono 2019; Sutanta 2012).

Government authorities, petrol station facilities and local communities, particularly those in surrounding developments, should be prepared to respond effectively to a fire emergency should it occur. Preparedness to respond to disasters includes engaging in various activities such as Emergency Operational Planning (EOP), exercises and drills, developing warning systems, public education, etc. (Coppola 2015; Qonono 2019). Therefore, this paper recommends that more research be conducted on the level of preparedness for disaster emergencies such as fires and explosions at petrol station facilities.

## Conclusion

Petrol station facilities contain large quantities of hazardous materials with a high risk of fire or explosion that could negatively impact people and the environment. Existing cases of fire and explosions at petrol station facilities in various parts of the world have proven this to be true. Handling petroleum products, which includes installing petrol station facilities, should be taken with great care, and compliance with safety best practices should be ensured. However, economic benefits and convenience overlook safety concerns around the siting of petrol station facilities. Countries share similar concerns regarding public safety, environmental protection and proper urban planning but have varying levels of detail and approaches based on local contexts. Disaster risk reduction should be embedded in land-use development planning to protect people and their environment from hazardous materials and proactively manage risks rather than disasters.

The findings of this study, while focused on South Africa’s major urban centres, offer a spatially grounded methodology and policy-relevant insights that are transferable to other rapidly urbanising cities facing similar challenges. Implementing these insights in broader regional or international contexts – particularly in low- and middle-income countries – could contribute significantly to safer urban planning practices and more effective DRR frameworks.

### Study limitations

This study used GIS to identify, analyse and map petrol station facilities in Johannesburg, Cape Town and Durban. Compliance was assessed using the EIA guidelines requiring a 100-m buffer from sensitive sites and a 3-km spacing between stations. These standards apply to facilities developed post-2001. A key limitation is the reliance on desktop analysis without input from local authorities regarding approval processes. The focus remained on centrally located petrol stations within the urban cores of the three major cities.
